# Tablet-based Rey–Osterrieth Complex Figure copy task: a novel application to assess spatial, procedural, and kinematic aspects of drawing in children

**DOI:** 10.1038/s41598-024-67076-9

**Published:** 2024-07-22

**Authors:** Marta Frigeni, Marco A. Petilli, Silvia Gobbo, Valentina Di Giusto, Carla F. Zorzi, Marco Rabuffetti, Federico Spinelli, Valerio Gower, Roberta Daini, Anna Cavallini

**Affiliations:** 1 Fondazione Don Carlo Gnocchi IRCCS S. Maria Nascente, Milan, Italy; 2 Division of Medical Genetics, Department of Pediatrics, Northwell Health, Great Neck, NY USA; 3 Department of Psychology, Università Degli Studi Di Milano-Bicocca, Milan, Italy

**Keywords:** Rey–Osterrieth figure, Movement control, Organizational strategies, Visuo-constructional skills, Tablet-based neuropsychological tests, Cognitive neuroscience, Computational neuroscience, Psychology, Neurology

## Abstract

The paper-and-pencil Rey–Osterrieth Complex Figure (ROCF) copy task has been extensively used to assess visuo-constructional skills in children and adults. The scoring systems utilized in clinical practice provide an integrated evaluation of the drawing process, without differentiating between its visuo-constructional, organizational, and motor components. Here, a tablet-based ROCF copy task capable of providing a quantitative assessment of the drawing process, differentiating between visuo-constructional, organizational, and motor skills, is trialed in 94 healthy children, between 7 and 11 years of age. Through previously validated algorithms, 12 indices of performance in the ROCF copy task were obtained for each child. Principal component analysis of the 12 indices identified spatial, procedural, and kinematic components as distinct dimensions of the drawing process. A composite score for each dimension was determined, and correlation analysis between composite scores and conventional paper-and-pencil measures of visuo-constructional, procedural, and motor skills performed. The results obtained confirmed that the constructional, organizational, and motor dimensions underlie complex figure drawing in children; and that each dimension can be measured by a unique composite score. In addition, the composite scores here obtained from children were compared with previsions results from adults, offering a novel insight into how the interplay between the three dimensions of drawing evolves with age.

## Introduction

Graphomotor skills comprise a subset of fine motor skills referring to the manual operation of a pencil or pen, typically during handwriting or drawing^1^. The term graphomotor skills is often used interchangeably with handwriting skills^1,2^; however, handwriting involves the cognitive knowledge of letters and words in addition to graphomotor skills^1^. Graphomotor tasks constitute up to 60% of daily activities in school and kindergarten^3,4^; and children with graphomotor deficits often show lower academic performance than their peers^5^. Identifying graphomotor problems at the earliest possible stage is crucial and can guide intervention to prevent learning difficulties later in life^5^. Several graphomotor evaluation tools are available for children; however, they assess either quality or speed of performance, not taking into account many of the components involved in executing graphomotor tasks^6^.

Recently, a shift from a product-oriented to a process-oriented assessment of the constructional, organizational, and motor abilities required to execute graphomotor tasks has occurred. In this scenario, the introduction of digital boards, such as tablets equipped with pens, has enabled the numerical analysis of dynamic and kinetic aspects of graphomotor tasks^7^. Tablet-based digital systems allow to obtain a variety of indices of performance that capture the three fundamental dimensions underlying the execution of graphomotor tasks^8–12^: the spatial dimension provides information on a subject’s visuo-constructional skills, and precisely the success in forming two-dimensional figures; the procedural dimension assesses a subject’s ability to use perceptual organization strategies; lastly, the kinematic dimension captures aspects of movement control that are crucial for handwriting and drawing. There are several studies assessing graphomotor skills using a tablet-based method, but they mainly focus on handwriting abilities^7,11,13^; by contrast, fewer studies have assessed drawing abilities using a tablet-based method^8–10,14^.

**Figure 1:**
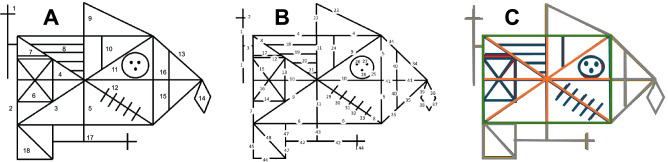
Division of the Rey–Osterrieth Complex Figure into: (**A**) 18 geometrical units according to the classical classification^17^; (**B**) 48 basic elements (44 single segments, 3 dots, and 1 circle); (**C**) 4 constitutive structures^49^ (base rectangle in green, main substructure in orange, outer configuration in grey, inner details in dark blue; the base rectangle and the main substructure represent the most important organizational units and the guiding structures when copying the Rey–Osterrieth Complex Figure). The figure is a reproduction of the original created using Microsoft PowerPoint (Microsoft 365; Version 16.71; www.office.com).

Drawing can be defined as the ability of combining one-dimensional units to form two-dimensional models; thus, it is a direct measure of a subject’s visuo-constructional skills. For more than 60 years, complex figure drawings have been utilized as a measure of visuo-constructional skills^15^. Among them is the Rey–Osterrieth Complex Figure (ROCF) copy task, first designed by Swiss neuropsychologist André Rey as an assessment tool for measuring visuo-constructional skills and memory in adults with brain damage^16,17^. This test consists in reproducing the 18 geometrical units ^17^ that constitute the ROCF by copying it freehand (Fig. 1A).

Since its initial introduction, the ROCF copy task has been extensively used to assess visuo-constructional skills, perceptual organizational strategies, as well as memory in adults and children^16–20^. In addition, this task has demonstrated the ability to distinguish between control groups and pediatric and adult patients with a wide variety of disorders, suggesting that it requires the integration of multiple processes^18–29^.

Several scoring methods have been developed to assess the performance in the ROCF copy task. The Rey–Osterrieth 36-Point Accuracy Scoring Method is the most widely used in clinical practice: it qualitatively determines the accuracy in drawing each of the 18 units constituting the ROCF (Fig. 1A) and their relative position compared to the original design presented^17,30–34^. Each unit is given a score from 0 (lowest) to 2 (highest) based on copy accuracy compared to the original design. This method has several merits, including adequate internal consistency ^35,36^ and test–retest reliability^37,38^. In addition, the Rey–Osterrieth 36-Point Accuracy Scoring Method has a strong positive correlation with the performance in other visuospatial perceptual tests, such as Visual Reproduction of the Wechsler Memory Scale^35^, line orientation^35^, and Raven's Standard Progressive Matrices^39^. However, this method doesn’t provide information on perceptual organization strategies^21^. Several alternative scoring systems have been proposed, including the Boston Qualitative Scoring System^40^, the system developed by Meyers and Meyers^38^, the Savage Scoring System^41,42^ that can assess perceptual organization strategies, and the Developmental Scoring System^43^; although the correlations obtained vary in strength and by task^22^. There is no single method capable of providing a comprehensive evaluation while also differentiating between the visuo-constructional, organizational, and motor components required for this copy task; and using more than one scoring method to differentiate between its components might not be routinely done in the clinical practice.

In this scenario, a tablet-based system could provide a more targeted account of a subject’s performance in the ROCF copy task. A tablet-based assessment of the ROCF copy task has been implemented and validated in healthy adults: this system allowed to accurately assess the temporal and kinematic aspects of the drawing process, as well as visuo-constructional accuracy, through the use of specific indices of performance^8^. Similar tablet-based approaches, effectively applied for other purposes or to other drawing tasks, have also been reported^11,12,29,44,45^.

The primary aim of this study was to apply the tablet-based assessment of the ROCF copy task previously validated only in adults^8^ to healthy elementary school children. In particular, it was hypothesized that this system would support the computation of unique and specific indices of performance in the pediatric population; and that three composite scores, each one underlying one of the three dimensions of the drawing process, could be derived from these indices through principal component analysis, as described in adults^8^.

An additional goal of this study was to analyze the relationship between each composite score and standardized paper-and-pencil measures of complex figure drawing in children. It was hypothesized that composite scores would correlate with corresponding paper-and-pencil measures of the visuo-constructional, organizational, and motor skills required for complex figure drawing in children.

Lastly, the performances of healthy children and adults in this tablet-based ROCF copy task system were compared^8^. The expectation was to identify similarities between children and adults, with one exception: it was hypothesized that age would positively correlate with ROCF copy task performance in children, as compared to adults where aging can be associated with a declining performance.

## Methods

### Study participants

Study participants were recruited from an elementary school located in the Lombardy region of Northern Italy. To be included in the study, each participant had to meet the following inclusion criteria: (i) age ranging from 7 to 11 years; (ii) lack of neurologic, neuropsychological, or neuropsychiatric disorders, as reported by either parents or teachers; (iii) normal or corrected-to-normal vision as reported by either parents or teachers; and (iv) not having previously completed the ROCF copy task. Prior to testing, informed consent was obtained from a parent or legal guardian. The study was approved by the Local Ethic Committee of Don Gnocchi Foundation IRCCS Santa Maria Nascente (Milan, Italy), and performed in accordance with the Helsinki Declaration.

A total of 98 participants met inclusion criteria, but 4/98 experienced technical difficulties that didn’t allow recording of the ROCF copy task on the graphic tablet. Since repeating the copy task was not permitted based on the study inclusion criteria, ultimately 94 participants were included in this study (Table 1). Mean age was 9.0 years (± 1.3 years). Participants’ gender and mean level of parental education have been previously shown not to exert any significant effect on ROCF copy task performance^18^. Each participant used their dominant hand to perform the ROCF copy task^46^.

**Table 1:** In the second column the number (N) of study participants is reported for each grade and overall; the third column reports a descriptive statistics (mean and standard deviation (SD)) of the study participants’ age for each grade and overall.

Study participants	N	Mean age in years (± SD)
2nd grade	20	7.5 (± 0.2)
3rd grade	31	8.4 (± 0.2)
4th grade	22	9.6 (± 0.2)
5th grade	21	10.5 (± 0.3)
Total	94	9.0 (± 1.3)

### Procedure

Each study participant was individually tested in a quiet room. Participants were asked to sit on a chair with a graphic tablet (Wacom Intuos Pro, Germany) placed in front of them. The tablet was equipped with a dedicated ballpoint pen (Ink Pen, Wacom, Germany); and it was wirelessly connected to a laptop computer running on Windows10 and operated by a single experienced examiner. Each participant was asked to copy the ROCF on a A4 size blank paper covering the entire active area of the tablet as accurately as possible, using the dedicated ballpoint pen just like in the conventional paper-and-pencil test^16,17^; no time limits were given^16,17^. This allowed to virtually recreate the same texture conditions that characterize the conventional paper-and-pencil method; and also to obtain ROCF copy task conventional scores using the 36-Point Accuracy Scoring Method^31^, as well as ROCF copy strategy scores using the Savage Scoring System^41,42^. The reference ROCF was available on a A4 size paper placed next to the tablet. The pen was wirelessly connected to the graphic tablet that was capturing every pen-down event (i.e., pen in contact with the drawing surface of the paper) and recording the pen-tip position compared to the paper’s surface at a rate of 100 Hz with a precision of 0.3 mm. The entire drawing process from the first to the last pen-down event was recorded, and the pen-tip position at any pen-down event was measured over time. Compared to the conventional paper-and-pencil method, this digital system allows to simultaneously assess visuo-constructional skills, perceptual organization strategies, and the degree of movement control involved in the drawing task^8,12,13,44,45^.

In addition, each study participant was asked to perform the Beery-Buktenica Test of Visual-Motor Integration^47^ (Beery VMI) and the Standardized Italian Test of Handwriting and Orthographic Competences^48^ (BVSCO-3) using conventional paper-and-pencil methods. It has to be noted that 2/94 study participants didn’t complete the Beery VMI and BVSCO-3 batteries; when working with young children in a school, sometimes there are unforeseeable circumstances that prevent a child from completing a test battery, as simple as a child having to leave earlier. The Beery VMI is a validated test^47^ commonly used in clinical practice as a measure of visual-constructional abilities in children. Thus, the study participants’ performance in the Beery VMI was used as an internal control of their normal visuo-constructional skills; and it was compared with the tablet-derived measure of visuo-constructional skills in order to assess its validity. Similarly, the BVSCO-3 is a validated test composed of three batteries^48^ that is routinely used to assess hand-motor skills in Italian-speaking children; it was used here as an internal control of the participants’ normal motor skills, as well as a measure of the tablet-derived kinematic score’s validity.

All paper-and-pencil tests were administered and scored by an experienced neuropsychologist (Table 2).

**Table 2:** Descriptive statistics (mean and standard deviation (SD)) and synthetic description of the digital ROCF copy task indices; the ROCF paper-and-pencil scores using the 36-Point Accuracy Scoring Method^31^ and the Savage Scoring System^41,42^; and the Beery VMI^47^ as well as BVSCO-3^48^ scores using conventional paper-and-pencil methods. The digital ROCF copy task indices were labeled with a lowercase letter “s” for spatial indices, “p” for procedural indices, and “k” for kinematic indices. sHP: horizontal placement accuracy index. sVP: vertical placement accuracy index. sLG: length accuracy index. sIC: inclination accuracy index. pBR: base rectangle priority index. pID: inner details priority index. pOR: organization by relevance index. pFR: fragmentation index. kVL: mean velocity index. kAC: mean acceleration index. kDC: mean deceleration index. kPK: number of peak velocity index. Scoring ranges are reported within parentheses, with 0 + representing a range with no predefined maximum value.

	Mean score	SD	Description
INDEX: sHP	5.2 (range 0–0 +)	2.6	Higher sHP = lower accuracy in positioning basic elements of ROCF on the horizontal axes
INDEX: sVP	4.6 (range 0–0 +)	2.1	Higher sVP = lower accuracy in positioning basic elements of ROCF on the vertical axes
INDEX: sLG	7.7 (range 0–0 +)	2.9	Higher sLG = lower accuracy in reproducing the length of the basic elements of the ROCF
INDEX: sIC	9.0 (range 0–0 +)	3.3	Higher sIC = lower accuracy in reproducing the inclination of the basic elements of the ROCF
INDEX: pOR	15.9 (range 0–0 +)	5.9	Higher pOR = lower level of organization for the basic elements of the ROCF
INDEX: pBR	37.4 (range 0–100)	12.1	Higher pBR = lower level of priority given to the base rectangle of the ROCF
INDEX: pID	70.1 (range 0–100)	13.0	Higher pID = lower level of priority given to the inner details of the ROCF
INDEX: pFR	13.1 (range 0–0 +)	5.9	Higher pFR = higher degree of fragmentation of the geometrical units of the ROCF
INDEX: kVL	30.2 (range 0–0 +)	6.5	Higher kVL = higher mean velocity per stroke
INDEX: kAC	208.8 (range 0–0 +)	59.7	Higher kAC = higher change of rate of velocity during acceleration phases
INDEX: kDC	193.5 (range 0–0 +)	61.0	Higher kDC = higher change of rate of velocity during deceleration phases
INDEX: kPK	5.1 (range 0–0 +)	1.1	Higher kPK = higher number of peaks per stroke
ROCF copy accuracy	26.4 (range 0–36)	4.4	Higher scores = higher copy accuracy
ROCF copy strategy	1.9 (range 0–6)	1.4	Higher scores = higher ROCF copy strategy
Beery VMI	19.5 (range 1–24)	2.2	Higher score = higher visual-constructional abilities
BVSCO-3 (3 batteries)	59 (range 0–0 +)	14.1	Higher score = higher motor control
	54.67 (range 0–0 +)	17.5	
	60.68 (range 0–0 +)	23.7	

### Tablet-based ROCF copy task data analysis

**Figure 2:**
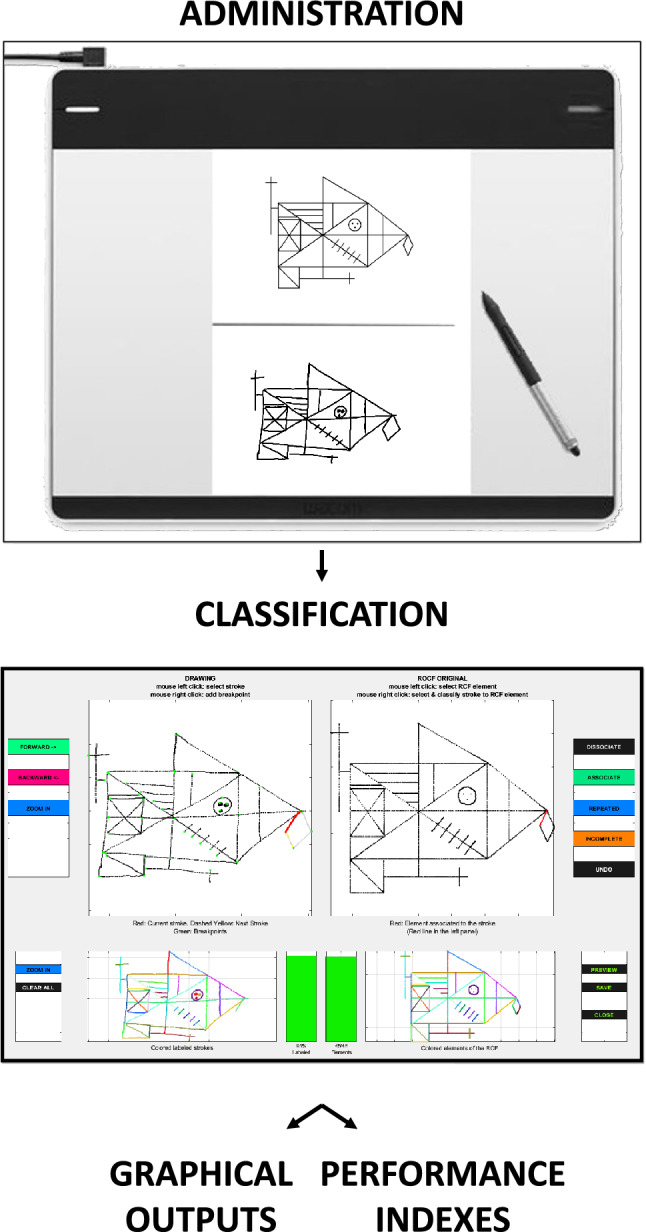
Schematic representation of four main stages of data analysis: administration stage; classification stage; pre-processing stage (not shown); graphical output and performance indexes stage. The figures are created using MATLAB version 2017b (MathWorks, Natick, MA, USA, www.mathworks.com) and Microsoft PowerPoint (Microsoft 365; Version 16.71; www.office.com).

In order to acquire and analyze data from each study participant’s performance in the tablet-based ROCF copy task, a MATLAB (MathWorks, Natick, MA, USA; www.mathworks.com) software previously described and validated was utilized^8^. The software employs functions from the following MathWorks toolboxes: Signal Processing Toolbox (Version 7.5), Optimization Toolbox (Version 8.0), Statistics and Machine Learning Toolbox (Version 11.2), and Image Processing Toolbox (Version 10.1). Data analysis was carried out following four main stages (Fig. 2), as previously described^8^: administration stage; classification stage; pre-processing stage; graphical outputs and performance indices stage. While the first stage can take a variable amount of time depending on how fast the ROCF copy task is completed, the following three stages can take a minimum of 5 min to be completed by a trained operator.

During the administration stage, the entire drawing process from the first to the last pen-down event was recorded using the graphic tablet that was connected to a computer. In the classification stage, a semi-manual classification of the segmented strokes into the 48 basic elements of the ROCF (Fig. 1B) was performed, as previously described^8^. The digital recordings of the ROCF drawings were transformed in the pre-processing stage in order to eliminate global distortion of the copy outputs^8^. In the last stage, three graphical outputs for each study participant were generated: a graphic output of the ROCF drawn; a velocity output with a visual representation of the velocity profile in drawing the ROCF; and a procedural output of the drawing sequence of each ROCF’s basic element. Additionally, during the last stage the analysis software provided 12 indices of performance for each study participant. These indices, that were previously demonstrated to capture the spatial, procedural, and kinematic dimensions underlying the execution of the ROCF copy task in adults^8^, are presented in the following paragraphs.

The spatial indices provided information on a participant’s ability to accurately reproduce the basic elements that constitute the ROCF and included: the horizontal placement accuracy (sHP) index; the vertical placement accuracy (sVP) index; the length accuracy (sLG) index; and the inclination accuracy (sIC) index. Each index was calculated as a measure of the deviation from the reference placement (mean) of each ROCF’s basic element. The accuracy in reproducing the spatial arrangement of the ROCF's basic elements decreased, as the deviation from the reference range increased. sHP and sVP indicated the level of precision in maintaining the horizontal and vertical placement of the ROCF's basic elements, respectively; sLG represented the degree of accuracy in preserving the relative size of each ROCF’s basic element compared to the others; sIC represented the degree of accuracy in preserving the relative inclination of each ROCF’s basic elements compared to the others.

The procedural indices provided information on participants’ constructional strategies and included: base rectangle priority (pBR) index; inner details priority (pID) index; organization by relevance (pOR) index; fragmentation (pFR) index. Altogether the procedural indices measured the ability to use an organized strategy when copying the ROCF. The ROCF is made of 4 constitutive structures^49^: the base rectangle (Fig. 1C, in green), the main substructure (Fig. 1C, in orange), the outer configuration (Fig. 1C, in grey), and the inner details (Fig. 1C, in dark blue). The base rectangle and the main substructure represent the most important organizational units and the guiding structures when copying the ROCF. Thus, highest scores were given when the ROCF copy started from its constitutive elements, namely the base rectangle and the main substructure, and ended with its secondary elements, namely the inner details and the outer configuration (Fig. 1C)^8,21^. Specifically, the pBR and pID indices measured the level of priority given to the base rectangle and to the inner details, respectively, on a scale from 0 to 100; 0 represented the first pen-surface contact, while 100 represented the last contact. Subsequently, the higher the pBR or pID, the lower the priority given. The pOR index measured the number of times a participant interrupted drawing a constitutive element of the ROCF and proceeded to draw a secondary element. The higher the pOR, the higher the number of interruptions. The pFR index measured the number of times a participant interrupted drawing a basic element of the ROCF to start another element. The higher the pFR, the higher the number of interruptions and therefore fragmentation of the ROCF’s basic elements.

Kinematic indices were obtained for the entire drawing process, from the first to the last pen-down event, using a method previously described^8^. Each subject’s velocity profile was smoothed using a fourth-order Butterworth filter with a cut-off of *7* Hz^8^. A pen-down event between two velocity throughs (< 5 mm/s) of 5 mm/s or less was considered a pen-stroke; only pen-strokes longer than 10 mm were included in the analysis and used to extract kinematic indices^8^. The kinematic indices provided information on participants’ movement control and included: mean velocity (kVL) index; mean acceleration (kAC) index; mean deceleration (kDC) index; and number of peak velocity (kPK) index. The kVL index provided information on pen-tip’s position changes over time; the higher the kVL, the higher the participant’s movement control. The kAC and kDC indices measured velocity changes during acceleration and deceleration, respectively. Specifically, these indices measured the mean acceleration per stroke and the average velocity change across all strokes. The higher the kAC and kDC, the higher the movement control. The kPK index measured movements’ fluency by averaging the number of velocity peaks across all pen strokes. The velocity profile of a perfectly fluent stroke is characterized by a single velocity peak. The higher the number of velocity peaks, the lower the movements fluency.

The mean and standard deviation for each index in the study participants is summarized in Table 2.

### Statistical analysis

For each of the 94 study participants, 12 indices of performance were obtained from the tablet-based ROCF copy task. For comparability, the same analyses previously conducted in adults using the same graphic tablet and data analysis software (i.e., R studio, version 4.3.2) were applied^8^. In order to reduce the set of indices into a smaller set of composite scores, the indices were standardized and Principal Component Analysis (PCA) with orthogonal Varimax rotation of the loading matrix applied^50–53^; subsequently, Kaiser–Meyer–Olkin Measure of Sampling Adequacy^54^ was performed to examine the appropriateness of the datasets for PCA, as previously described^8^. The communalities of each index were evaluated, with items poorly accounted for by the factor solution indicated by a communality below 0.5. Primary loadings were deemed meaningful if they exceeded 0.50; while indices were considered as cross-loading indices if their loadings exceeded ± 0.30 on two or more components. In order to simplify the model structure, cross-loading indices were not retained. A cumulative scale constructed by averaging the indices’ loadings for the spatial, procedural, and kinematic components, respectively, was used to extract a composite score for each component. Before creating the composite scores, negative loadings were reverse scored. The reliability of the cumulative scales was measured by Cronbach’s alpha. Values that exceed 0.70 were deemed above the recommended level of acceptability. Composite scores were subsequently correlated with conventional paper-and-pencil measures of ROCF copy accuracy (as measured by the 36-Point Accuracy Scoring Method^31^), ROCF copy strategy (as measured by the Savage Scoring System^41,42^), visuo-constructional skills (as measured by Beery VMI^47^), and motor skills (as measured by BVSCO-3^48^), as well as participants’ age. In regard to participants’ age, it has to be noted that children with a normal neurodevelopment attend grades based on their chronological age. Since children in the same grade are approximately the same age, age and educational level can be considered overlapping in the pediatric population here presented. By contrast, age and educational level don’t overlap in adults, where a higher level of education is generally associated with a higher ROCF copy task performance^8^, while higher age is associated with a decline in performance due physiological aging^8^.

## Results

### Principal component analysis

**Figure 3:**
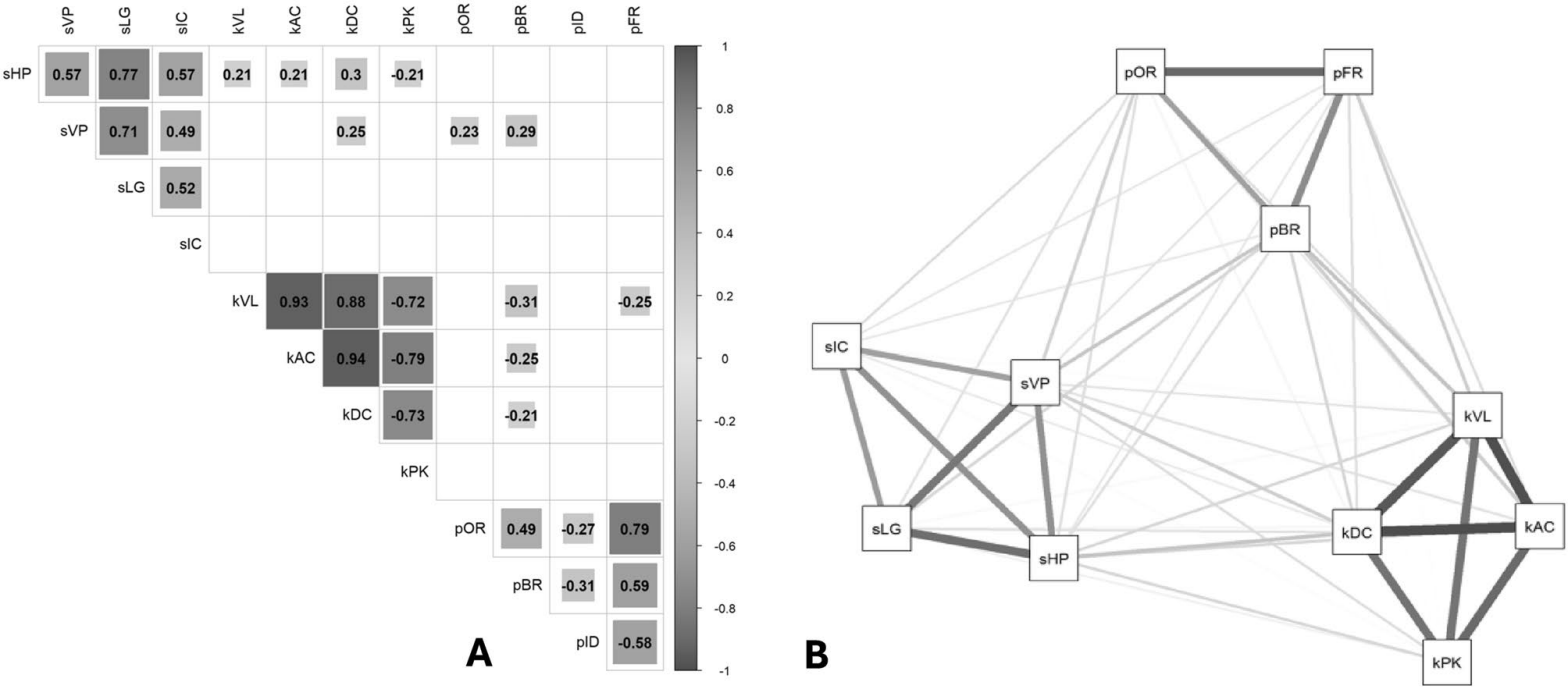
Correlations among the indices. (3A) Significant correlations (p < 0.05) are reported in greyscale, with highest correlations in dark grey. (3B) Visual representation of the patterns of correlations among 11 indices after exclusion of pID. The figures were created in RStudio (Version 1.4.1106) with the package corrplot (version 0.88).

The minimum amount of data for PCA analysis was met, with a sample size of 94 study participants and a calculated ratio of 7.9 observations per variable^53^. PCA was deemed appropriate in reducing the original 12 indices into a smaller number of composite scores, based on the following three criteria: a level of correlation between two or more variables of at least 0.3, suggesting a high level of redundancy in the data (Fig. 3A); a significant correlation among several indices, as shown by the statistically significant result of Bartlett’s test of sphericity^55^ (χ^2^(55) = 853.06, p < 0.001); and lastly, a minimum MSA level of 0.5^53^. Only the MSA for pID was slightly below the accepted level (pID MSA: 0.48), in line with the analysis performed in healthy adults^8^. Ultimately, pID exclusion from the analysis resulted in an overall MSA level of 0.75, with a minimum MSA for individuals indices of 0.58 (Fig. 3B). PCA was then performed on the 11 remaining indices; and Horn’s parallel analysis for PCA indicated the existence of three components, in line with previous assumptions^8^ (observed eigenvalues: C1 = 3.84, C2 = 3.15, and C3 = 1.73; simulated eigenvalues: C1 = 1.59, C2 = 1.41, and C3 = 1.28). The observed eigenvalues for the first three components were significantly higher than those for the simulated randomly generated datasets (ΔC1 = 2.25; ΔC2 = 1.74; ΔC3 = 0.45). The Kaiser's criterion^54^ was used to confirm the existence of three components, that together accounted for 79.3% of the variance in total scores. Specifically, the first three components explained 34.9%, 28.6%, and 15.7% of the variance, respectively.

**Figure 4:**
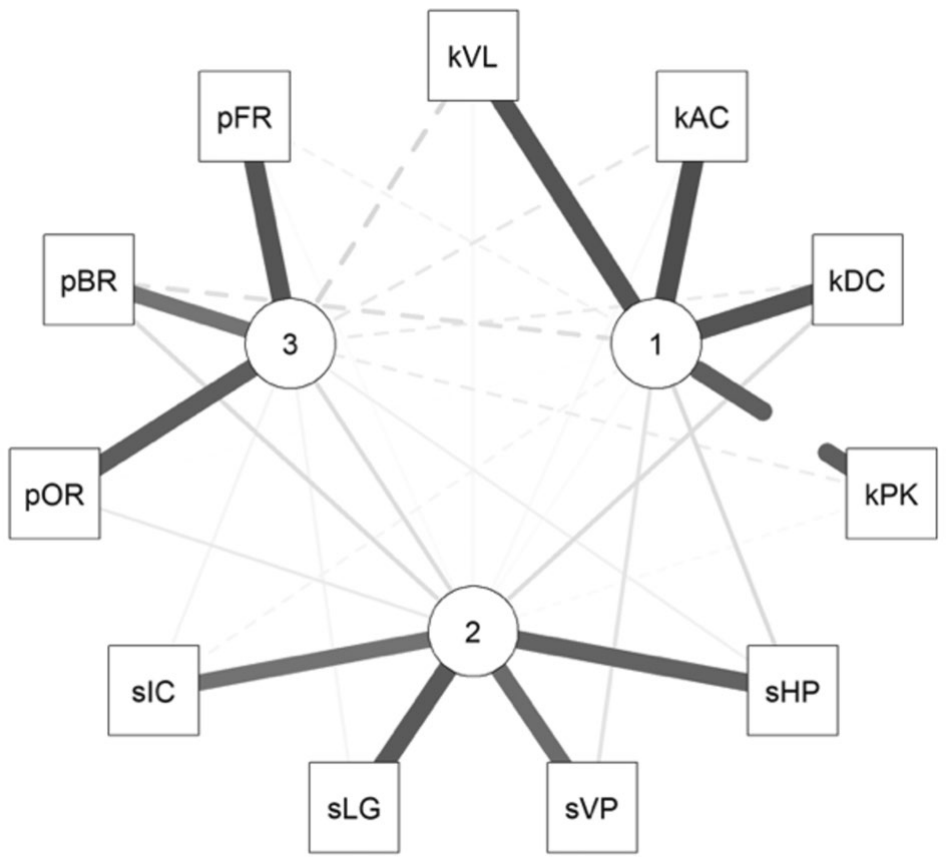
Visual representation of the correlations between the 11 indices and how they grouped into three separate components based on the PCA. The three components are indicated by numbered nodes (1, 2, and 3): the nodes are located closer to the indices they demonstrated higher correlation with. The figure was created in RStudio (Version 1.4.1106) with the package corrplot (version 0.88).

The component matrix results highlighted commonalities for all indices higher than 0.5, with no index exhibiting cross-loading (criteria for cross-loading: two loadings greater than ± 0.30). With primary loadings greater than ± 0.50 for each of the indices, and a combined loading greater than ± 0.70, the PCA confirmed its very robust structure. Standardized loadings (pattern matrix) based upon the correlation matrix for the three components (RC1, RC2, and RC3 for 1, 2, and 3, respectively) are shown in Table 3. Overall, the structure of relationships between the various dimensions of drawing overlapped what previously described in healthy adults^8^ (Fig. 4 and Table 3).

**Table 3:** Standardized loadings (pattern matrix) derived from the correlation matrix for the three components (RC1, RC2, and RC3), obtained through Principal Component Analysis with orthogonal Varimax rotation of the loading matrix are reported (see main text for further details). Primary loadings greater than ± 0.50 for each component are shown in bold. The component communalities (i.e., the proportion of variance in each index variable that is accounted for by the components) are shown in column “h2”. Each component's uniqueness (i.e., the amount of variance in each variable not explained by the components, or 1–h2) are shown in column "u2". Hoffman's index of complexity for each index variable (i.e., the number of latent components required to account for the variance observed in those variables) are shown in column "com".

	RC1	RC2	RC3	h2	u2	com
sHP	0.19	**0.85**	0.08	0.77	0.23	1.10
sVP	0.14	**0.80**	0.18	0.69	0.31	1.20
sLG	0.03	**0.90**	0.06	0.82	0.18	1.00
sIC	− 0.06	**0.76**	0.06	0.59	0.41	1.00
kVL	**0.93**	0.05	− 0.21	0.91	0.09	1.10
kAC	**0.97**	0.04	− 0.12	0.96	0.04	1.00
kDC	**0.93**	0.18	− 0.11	0.91	0.09	1.10
kPK	− **0.87**	− 0.04	− 0.10	0.78	0.22	1.00
pOR	− 0.01	0.11	**0.88**	0.78	0.22	1.00
pBR	− 0.17	0.18	**0.75**	0.63	0.37	1.20
pFR	− 0.07	0.03	**0.93**	0.87	0.13	1.00

The first component (RC1) showed significant positive primary loadings for kVL, kAC, kDC, but a significant negative loading for kPK. This composite score, renamed “Kinematic” (KIN), provided information on the kinematic aspects of the ROCF copy task; lower values of KIN were associated with greater movement control. The second component (RC2) showed high positive primary loadings for sHP, sVP, sLG, and sIC. This composite score, renamed “Spatial” (SPA), provided information on the accuracy in reproducing the spatial relationships of the ROCF’s basic elements; lower values of SPA were associated with greater accuracy in reproducing the spatial relationship of the ROCF’s basic elements. Lastly, the third component (RC3), renamed "Procedural" (PRO), demonstrated positive primary loadings with pOR, pBR, and pFR, indicating that lower values of PRO were associated with better organizational strategy in drawing the ROCF. Of note, due to its negative loading the kPK index was reverse-scored prior to the creation of the KIN composite score. The SPA and the PRO composite scores were also reversed scored so that higher scores represented better performances for all the composite measures. The assumption of dimensionality for each composite score was supported by their clean interpretation in the model’s results (i.e., indices were strongly interrelated and represented a single component distinctly). For all scales, Cronbach’s alpha exceeded the recommended threshold of 0.70, indicating satisfactory internal consistency (0.86 for the scale of SPA; 0.95 for the scale of the KIN; 0.83 for the scale of PRO).

Correlation analyses for the composite scores highlighted a significant correlation between SPA and PRO (r = 0.22; p = 0.03); by contrast, there was no significant correlation between SPA and KIN (r =  − 0.16; p = 0.12), and between PRO and KIN (r = 0.17; p = 0.10).

### Correlations between tablet-based ROCF copy task and conventional paper-and-pencil tests

As a final step in the analysis, the relationship between composite scores and age, as well as between composite scores and conventional paper-and-pencil measures of ROCF copy accuracy (as measured by the 36-Point Accuracy Scoring Method^31^), ROCF copy strategy (as measured by the Savage Scoring System^41,42^), visuo-constructional skills (as measured by Beery VMI^47^), and motor skills (as measured by BVSCO-3^48^) were assessed through partial correlation analysis using Pearson correlation coefficient. All statistical analyses were conducted through the program R (version 4.3.1) and the packages *psych*^56^*, dplyr*^57^*, corrplot*^58^*, qgraph*^59^*, ltm*^60^*.* Correlation results are presented in Table 4.

**Table 4:** Correlation coefficient (r) between each composite score (SPA, PRO, and KIN) obtained from the performance in the tablet-based ROCF copy task and study participants’ age as well as paper-and-pencil ROCF copy accuracy, ROCF copy strategy, Beery VMI, and BVSCO-3. Statistically significant p values are reported with an asterisk: *p < 0.05; **p < 0.01; ***p < 0.001. A correlation coefficient of 0.10 represented a weak or small association^62^; a correlation coefficient of 0.30 was considered a moderate correlation^62^; and a correlation coefficient of 0.50 or larger represented a strong or large correlation^62^ (bold r values).

SPA	AGE (N = 94)	ROCF copy accuracy (N = 94)	ROCF copy strategy (N = 94)	Beery VMI (N = 92^1^)	BVSCO-3^1^ (N = 92^2^)		
	r = 0.29**	r = 0.77***	r = 0.04	r = 0.33**	r = 0.04	r = − 0.07	r = 0.08
PRO	r = 0.16	r = 0.25*	r = 0.64***	r = 0.18	r = − 0.09	r = − 0.14	r = − 0.15
KIN	r = 0.19	r = − 0.17	r = 0.10	r = − 0.23*	r = 0.12	r = 0.00	r = 0.17

^1^Scores obtained from participants’ performance in the three paper-and-pencil tests that are part of the BVSCO-3 battery are reported.

^2^For 92/94 study participants, performance in the conventional paper-and-pencil Beery VMI and BVSCO-3 tests was compared with each composite score (SPA, PRO, and KIN).

SPA demonstrated a positive and significant correlation with age (r = 0.29; p < 0.01); similarly, there was a positive although not significant trend between KIN and age (r = 0.19; p = 0.06). By contrast, there was no significant correlation between PRO and age (r = 0.16; p = 0.11). Both SPA (r = 0.77; p < 0.001) and PRO (r = 0.25; p < 0.05) showed a positive and significant correlation with paper-and-pencil ROCF copy accuracy assessed using the conventional 36-Point Accuracy Scoring Method^31^; while KIN did not correlate significantly with it (r = − 0.17; p = 0.11). Paper-and-pencil ROCF copy strategy assessed with the conventional Savage Scoring System^41,42^ correlated significantly with PRO (r = 0.64; p < 0.001), but not with SPA (r = 0.04; p = 0.70) or KIN (r = 0.10; p = 0.35). The performance in the Beery VMI^47^ demonstrated a positive and significant correlation with SPA (r = 0.29; p < 0.01), a negative and significant correlation with KIN (r = − 0.23; p < 0.05), but no significant correlation with PRO (r = 0.18; p = 0.09). Lastly, the performance in the BVSCO-3^48^ did not correlate with any of the three composite scores, nor demonstrated a trend toward any of them.

## Discussion

The use of a tablet-based digital system for analyzing graphomotor functions has several advantages compared to conventional paper-and-pencil methods. Through the computation of three composite scores, this system allows to simultaneously capture and differentiate the spatial, procedural, and kinematic dimensions of the drawing process^8,12,13,44,45^. The spatial dimension provides information on a subject’s visuo-constructional skills, and precisely the success in forming a two-dimensional figure; the procedural dimension provides information on a subject’s use of perceptual organization strategies; lastly, the kinematic dimension allows to capture aspects of movement control in drawing. These three separate dimensions cannot be derived from conventional paper-and-pencil scoring methods, such as the Rey–Osterrieth 36-Point Accuracy Scoring Method. Even when larger paper-and-pencil testing batteries are performed in order to provide an evaluation of all three components, the process is laborious and time-consuming in both the administration and evaluation stages.

The tablet-based ROCF copy task system here proposed and trialed for the first time in healthy children demonstrated to be a tool capable of comprehensively assess visuo-constructional, procedural, and motor skills, while also differentiating between these three components. The PCA’s results here presented in a pediatric population further support the use of separate scores to assess each dimension of the drawing process in the ROCF copy task (Figs. 3B and 4): SPA for the spatial dimension; PRO for the procedural dimension; and KIN for the kinematic dimension. Interestingly, the PCA results highlighted how the spatial and procedural dimensions of the drawing process have a higher degree of independence from each other in healthy children (Fig. 3B); by contrast, a greater interplay between these two components has been described in adults^8^. This phenomenon can be at least partially explained by an acquired integration between visuo-constructional skills and perceptual organization strategies, represented by SPA and PRO respectively, that is present in adults but has yet to develop in children. In adults, it has been postulated that the integration between SPA and PRO allows to adopt effective procedural strategies, with the goal of simplifying the constructional process^8^.

In order to provide further insights into the aspects underlying complex figures drawing in healthy children, as captured by the composite scores SPA, PRO, and KIN, correlation analyses were performed. Each composite score was initially correlated with participants’ age. Age showed a positive and significant correlation with SPA, indicating that visuo-constructional skills improve with age, as expected (Table 4). This is in contrast with what observed in adults, where the correlation between SPA and age is significant but negative, indicating that physiological aging is associated with a decline in visuo-constructional skills^8^. Age showed a positive although not significant trend toward KIN; this highlights how movement control physiologically improves with age, but eventually plateaus in adults^8^. There was no significant correlation between PRO and age, neither in the pediatric population here presented nor in adults^8^; while a positive correlation between PRO and educational level has been described in adults^8^. These findings suggest that PRO might correlate with cognitive changes associated to long-term education; thus, it’s postulated that perceptual organizational strategies are not yet fully developed during elementary school, and they consolidate later in the school years.

As a next step in the analysis, composite scores were correlated with conventional paper-and-pencil measures of ROCF copy accuracy (36-Point Accuracy Scoring Method^31^), ROCF copy strategy (Savage Scoring System^41,42^), visuo-constructional skills (Beery VMI^47^), and motor skills (BVSCO-3^48^). Higher SPA scores were associated with a better performance in conventional paper-and-pencil measures of spatial accuracy, namely the 36-Point Accuracy Scoring Method^31^ and the Beery VMI^47^. Of note, the highest correlation was observed between SPA and ROCF copy task accuracy (p < 0.001 vs p < 0.01 between SPA and Beery VMI): this is in line with what observed in adults^8^, further supporting the validity of SPA as a measure of visuo-constructional skills. The correlation between SPA and the other conventional paper-and-pencil measures of drawing were low and not significant. By contrast, PRO exhibited its highest correlation with organizational skills as measured by ROCF copy strategy using the Savage Scoring System^41,42^ (p < 0.001). In this regard, it has to be noted that PRO also correlated with ROCF copy accuracy (p < 0.05), although at a lower degree compared to ROCF copy strategy (p < 0.001). However, PRO did not show a significant correlation with the other measure of spatial accuracy considered in this study, namely the Beery VMI. This discrepancy can be attributed to a low specificity of the 36-Point Accuracy Scoring Method^31^, that is known to be influenced by perceptual organizational strategies in addition to visuo-constructional skills^61^. In contrast, the Beery VMI^47^ likely provides a purer measure of visuo-constructional skills, unaffected by organizational strategies. Lastly, KIN exhibited only a significant negative correlation with Beery VMI^47^ (p < 0.05). Previous research has shown a correlation between KIN and copy accuracy in drawing tasks administered to healthy adults; specifically, higher KIN was associated with a less meticulous and careful drawing style (lower spatial precision due to increased drawing velocity)^8^. A similar rationale may apply in the context of this study; in fact, the Beery VMI^47^ is a lengthy test, and can represent a challenge for elementary school children not used to keep a high level of concentration for a long period of time. Thus, it’s possible that the negative correlation between Beery VMI^47^ and KIN was dictated by the adoption of a rapid and careless drawing style, with subsequent negative repercussions on drawing accuracy. This pattern suggests that healthy individuals could trade off accuracy for a fastest execution time. Contrary to expectations, the BVSCO-3^48^ test battery did not correlate with the KIN or with any other composite score. The BVSCO-3^48^ is a paper-and-pencil Standardized Italian Test of Handwriting and Orthographic Competences, and was used here as an internal control to assess participants’ hand-motor skills. However, the test is influenced by orthographic competences in addition to hand-motor skills, and this contamination is most certainly responsible for the lack of correlation with the three composite scores.

Compared to conventional paper-and-pencil scoring methods that provide an integrated evaluation of the drawing process without differentiating between its components, the results presented here demonstrated that this tablet-based system is capable of extracting unique scores (SPA, PRO, and KIN) correlating with the skills required for drawing complex figures in healthy children. In this study 12 indices of performance were utilized and 3 composite scores derived from them, as previously described^8^. Nonetheless, the method here described has the potential of capturing additional indices of performance assessing other aspects of the drawing process. For instance, over thirty different temporal, kinematic, and dynamic indices examining handwriting movement using graphic tablets have been described^11^, as well as additional indices of visuo-constructive abilities for the early detection of cognitive impairment and dementia^12^. Considering different measures in the future could potentially improve the tools used in this field, and allow to determine which metrics better explain performance in the ROCF copy task in children.

Additional tablet-based system assessing the performance in the ROCF copy task have been developed over the years with different goals, including identifying a digital biomarker for Alzheimer disease^9^, and differentiating between healthy adults and patients with either mild cognitive impairment or dementia^29^. The authors speculate that this system could potentially identify and differentiate between deficits in specific areas of the drawing process in children, similarly to what has been reported for adults^29^. While additional studies in a patient population are required in order to prove this hypothesis, it’s important to note that being able to diagnose deficits in organizational, fine motor, and/or visuo-constructional skills could be used to build treatment plans tailored to patients’ specific needs.

Lastly, although the scoring algorithm here described is fully automated, the classification stage (Fig. 2) requires a semi-manual classification of the segmented strokes into the 48 basic elements of the ROCF (Fig. 1B). This process takes about 5 min per ROCF drawing. A novel automated classification algorithm capable of automatically identifying the drawing parts of a complex figure has been implemented^44^; thus, future studies could aim at integrating a similar algorithm into the tablet-based system here utilized, with the goal of cutting operator-depended analysis time and allowing to obtain almost instant results.

## Data Availability

The dataset generated and analyzed during the current study is available from the corresponding author upon request.
